# Cooperation, Competition, and Coalitions in Enzyme-Producing Microbes: Social Evolution and Nutrient Depolymerization Rates

**DOI:** 10.3389/fmicb.2012.00338

**Published:** 2012-09-27

**Authors:** Henry J. Folse, Steven D. Allison

**Affiliations:** ^1^Department of Ecology and Evolutionary Biology, University of CaliforniaIrvine, CA, USA; ^2^Department of Earth System Science, University of CaliforniaIrvine, CA, USA

**Keywords:** nutrient depolymerization, cooperation, spatial model, density-dependence, extracellular enzymes, facilitation, microbe, decomposition

## Abstract

Extracellular enzymes represent a public good for microbial communities, as they break down complex molecules into simple molecules that microbes can take up. These communities are vulnerable to cheating by microbes that do not produce enzymes, but benefit from those produced by others. However, extracellular enzymes are ubiquitous and play an important role in the depolymerization of nutrients. We developed a multi-genotype, multi-nutrient model of a community of exoenzyme-producing microbes, in order to investigate the relationship between diversity, social interactions, and nutrient depolymerization. We focused on coalitions between complementary types of microbes and their implications for spatial pattern formation and nutrient depolymerization. The model included polymers containing carbon, nitrogen, or phosphorus, and eight genotypes of bacteria, which produced different subsets of the three enzymes responsible for hydrolyzing these polymers. We allowed social dynamics to emerge from a mechanistic model of enzyme production, action, and diffusion. We found that diversity was maximized at high rates of either diffusion or enzyme production (but not both). Conditions favoring cheating also favored the emergence of coalitions. We characterized the spatial patterns formed by different interactions, showing that same-type cooperation leads to aggregation, but between-type cooperation leads to an interwoven, filamentous pattern. Contrary to expectations based on niche complementarity, we found that nutrient depolymerization declined with increasing diversity due to a negative competitive effect of coalitions on generalist producers, leading to less overall enzyme production. This decline in depolymerization was stronger for non-limiting nutrients in the system. This study shows that social interactions among microbes foraging for complementary resources can influence microbial diversity, microbial spatial distributions, and rates of nutrient depolymerization.

## Introduction

Microbial public goods are products that are secreted outside the cell, and therefore benefit not only the individuals producing them, but also neighboring cells (Velicer, 2003; West et al., 2006, 2007). They include substances crucial to intercellular interactions, such as quorum sensing molecules, biofilm polymers, siderophores, exoenzymes, and many other cell products (West et al., 2006, 2007). Public goods are ubiquitous in microbial ecosystems. However, evolutionary theory predicts that producers of public goods are vulnerable to cheating by individuals that receive the benefits without paying the cost of production. Exoenzyme production by bacteria and fungi is particularly important for ecosystem function because these enzymes catalyze the rate-limiting step in the depolymerization of carbon and nutrients from organic polymers in the environment (Schimel and Bennet, 2004). Thus the quantity and types of enzymes produced by microbes influence the rate at which these nutrients flow through the ecosystem. However, despite their importance to nutrient cycling, these enzymes have received little attention from a social evolution perspective relative to other public goods. At the same time, models of enzyme-mediated decomposition by microbes (for example Schimel and Weintraub, 2003) do not account for social interactions.

Allison (2005) applied a public goods framework to exoenzyme production by microbes and demonstrated that the presence of cheaters reduces nutrient depolymerization, and that the cost of cheating increases with the diffusion rate of the enzyme and the rate of constitutive enzyme production. This study considered an environment with one limiting nutrient and two types of microbes, producers, and cheaters. However, real microbial communities are highly diverse, with thousands of taxa (Roesch et al., 2007), and they depend on multiple nutrients. The present study extends the model of Allison (2005) to investigate the social dynamics of enzyme production in a multi-genotype community in a multi-nutrient environment.

We used this model to examine how diversity and social interactions modulate each other’s effects on nutrient depolymerization. Our first objective was to determine the conditions for the maintenance and loss of diversity. Second, in the context of social evolution, we determine the conditions under which coalitions can form and compete successfully. We define a coalition to be a mutualistic interaction between two or more complementary types that each produce enzymes lacking in the other. Since interactions due to diffusion occur locally, this would lead to greater growth and survival when complementary types are close together. We predict that complementary types should therefore be spatially associated and take such spatial correlation to be evidence for coalitions. Finally, in the context of ecosystem function, our third objective was to examine how diversity and coalitions affect rates of nutrient depolymerization. If social interactions limit the production of enzymes, these effects could cascade upwards, limiting the depolymerization of nutrients, and therefore the overall rate of flow through the ecosystem.

## Materials and Methods

### Model overview

The model is an individual-based, stochastic simulation coded in C++, built on a previous model by Allison (2005). It consists of a 100 × 100 lattice grid, where each grid box represents 1 μm^3^. In each box of the grid, the model tracks the substrate, enzyme, and product concentrations for each of the three nutrients, as well as the resident microbe if one is present. Each microbe has a genotype and a pool of nutrients internal to itself. At each time step of the model, the following processes occur in each grid box in this order: substrate input, substrate decay, product decay, product diffusion, enzyme decay, enzyme diffusion, product formation, nutrient uptake and enzyme production, microbial metabolism, death, and reproduction. Microbes optimize their nutrient uptake and enzyme production in order to balance their internal nutrients in their stoichiometric ratios.

The original study (Allison, 2005) analyzed the social dynamics of producers and cheaters in the case where only carbon was limiting. In the current model, carbon (C), nitrogen (N), and phosphorus (P) were all present only as substrates that must be hydrolyzed by enzymes in order to be available. We used a genetically explicit model with three loci, each of which coded for an enzyme that breaks down C, N, or P. At each locus, there was one allele for enzyme production and one for no enzyme production (i.e., cheating). In naming a genotype, we represent the former with the capital letter of the respective nutrient and the latter with the lowercase letter. Thus the genotype *CNp* represents a microbe that produces C- and N-enzymes, but not P-enzymes. There are eight genotypes in total: *cnp*, *Cnp*, *cNp*, *cnP*, *CNp*, *CnP*, *cNP*, and *CNP*. We also include mutation, which allows for the reintroduction of new types after they have been lost.

### Initialization

At the start of the model, the concentrations of substrate, enzyme, and product are all initialized to 0 over the entire grid. Each grid box may contain zero or one microbe, but no more than one microbe may occupy a single grid box. Thus the maximum density is 1 microbe/1 μm^3^, so the population is limited by space. Microbes are introduced randomly with a total frequency of 0.02, and each is assigned a random genotype with equal probability. The C biomass of each microbe is initialized to 150 fg C (Button et al., 1998), and the other nutrients within the microbe are initialized to maintain the stoichiometric ratios of C:N = 6 and C:P = 60.

### Iteration

Although conceptually the following processes occur simultaneously across all grid boxes, the program must compute them sequentially. The order of the grid boxes and of the nutrients is randomized so as to avoid bias, which would arise if the same order were used each time.

### Inputs

Substrate is added to each grid box at each time step, at rates of 0.1 fg C/min/μm^2^., 0.01 fg N/min/μm^2^, and 0.001 fg P/min/μm^2^. No product or enzyme is directly added to the grid.

### Decay and diffusion

The substrate, product, and enzymes were removed from the grid at a constant rate of 0.01/min. Substrate does not diffuse. Product diffusion rates are set to 0.5 μm^2^/min, meaning that the concentrations in two adjacent grid boxes will equilibrate in 1 min of model time. When diffusion occurs at a box, a random neighbor box is chosen, and an amount proportional to the difference in the concentrations of the two boxes is moved from the box with the higher concentration to that with the lower concentration. These values are based on diffusion and loss rates reported by Vetter et al. (1998).

### Product formation

Product is formed by the action of enzymes on substrate, following Michaelis–Menten kinetics:

$$\Delta \left[\text{Product}\right]=\left[\text{Enzyme}\right]*V_{\text{max}}*\frac{\left[\text{Substrate}\right]}{K_{m}+\left[\text{Substrate}\right]}*\Delta t$$

where *V*_max_ = 10 fg/fg/min is the maximum rate of product formation at substrate saturation, and *K*_m_ = 0.001 fg/μm^3^ is the half-saturation constant. These values fall within the range reported in the literature for hydrolytic enzymes (Schomburg and Schomburg, 2001). This quantity is also deducted from the substrate pool.

### Product uptake

Microbes only take up nutrients when they are in demand relative to the microbe’s stoichiometric ratios of C:N = 6 and C:P = 60. For example, if the microbe’s internal ratio is C:N > 6, this would mean that N was in demand, and the microbe would take up N in order to maintain its stoichiometric ratio. Although microbes are actually more flexible in their nutrient uptake, this constraint reflects a microbial tendency to maintain stoichiometric ratios within limits (Sterner and Elser, 2002).

The rate of product uptake is proportional to the surface area of the microbe and also follows the Michaelis–Menten kinetics:

$$\begin{array}{rcll} \Delta \left[\text{Nutrient}\right] & =\text{Enz}\;\text{Per}\;\text{Area}*\text{Area}\;\text{To}\;\text{Mass}*\text{Biomas}{\text{s}}^{\text{2/3}} & & \\ & \;*V_{\text{max}}*\frac{\left[\text{Product}\right]}{K_{m}\left[\text{Product}\right]}*\Delta t & & \end{array}$$

where Enz Per Area = 0.1 is the density of uptake enzymes on the exterior of the microbe, Area To Mass = 0.0428 is the ratio of surface area to volume of the microbe, *V*_max_ = 10 fg/fg/min is the maximum rate of uptake at product saturation, and *K_m_* = 0.001 fg/μm^3^ is the half-saturation constant. These values fall within the ranges reported by Button et al. (1998).

### Enzyme production

In each time step, if a microbe has the gene to produce an enzyme, it must produce the enzyme at a minimum constitutive rate, which by default is set to 10^−7^ fg/fg/min, times its biomass.

Facultative enzyme production occurs only if the nutrient is still in demand following nutrient uptake, in which case a maximum of 1% of uptake from the current time step is allocated to enzyme production. This value is within the range of 0.7–2.1% reported for α-glucosidase production by yeasts (Giuseppin et al., 1993) and slightly higher than the 0.3–0.9% reported for protease production by *Bacillus clausii* (Christiansen and Nielsen, 2002). Production of N and P-enzymes is initially calculated in units of N or P mass, respectively. This quantity is then converted to C mass using the stoichiometric ratios of C:N = 3.5 and C:P = 200. The first value is based on the stoichiometry of proteins, and the second on the assumption that small amounts of P could be lost during enzyme secretion, especially if protein phosphorylation is involved. When enzymes are produced, a quantity of C equal to 10% of enzyme C mass is respired due to the metabolic costs of enzyme production.

If producing enzyme at the maximum level will cause another nutrient to become limiting, the microbe will produce less enzyme. For example, the ratio of C:N for microbes is 6, nearly twice that of enzymes. Suppose initially the microbe’s C:N ratio is less than 6, indicating that C is limiting, and the microbe takes up a quantity of C equal to *uptake*. Then its maximum enzyme production will be 0.01 × *uptake*. However, producing this quantity may reduce the microbe’s N pool to the point where its C:N ratio is now greater than 6, making N limiting. In this case, the microbe will produce a quantity equal to

$$\text{Enz}\;\text{Prod}=\frac{\text{C}\;\text{to}\;{\text{N}}_{\text{mic}}*\text{N}-\text{C}}{\text{C}\;\text{to}\;{\text{N}}_{\text{mic}}∕\text{C}\;\text{to}\;{\text{N}}_{\text{enz}}-1-\text{Res}{\text{p}}_{\text{enz}}}$$

Here, C to N_mic_ is the microbial C:N ratio of 6, C to N_enz_ is the enzyme C:N ratio of 3.5, Resp_enz_ is the rate of respiration due to enzyme production, 0.1, and N and C are the current pools of N and C in the microbe. This quantity of enzyme production equalizes the microbe’s C:N ratio at its target level of C to N_mic_ = 6. Analogous calculations are applied to the other nutrient combinations.

### Microbial processes

In addition to product uptake and enzyme production, microbes also undergo metabolism, reproduction, and mortality. Microbes respire C at a constant basal metabolic rate (BMR), of 0.00015 fg/fg/min to account for cellular maintenance. This rate is 10 times higher than the range reported by Price and Sowers (2004) because we assume that actively growing microbes require more energy for maintenance metabolism. Thus the BMR also includes growth metabolism. Microbes also lose N and P in proportion to the amount of C lost by a factor of 0.1 divided by the C:nutrient ratio.

When a microbe reaches a critical mass of 300 fg, it divides, producing an exact copy of itself (unless mutation occurs). Mutations, which occur independently at each locus and change the allele to its complement, occur at a rate of 10^−5^/locus/division. One copy remains in the current grid box, and the other moves into a neighboring grid box. If that box is occupied, then one of the microbes dies randomly with probability 0.5.

Microbial mortality occurs randomly at a fixed rate of 3 × 10^−5^/min and also occurs if a microbe’s biomass falls below 30 fg C. This minimum mass is based on the low end of bacterial sizes reported by Button et al. (1998). When a microbe dies, its biomass and nutrients are added back to the grid, half as substrate and half as product.

For more details on the parameterization of the model, see Allison (2005).

### Model runs

We varied the enzyme diffusion rate (EDiff) over the values 10^−4^, 10^−3^, and 10^−2^, and the constitutive enzyme production rate (EConstit) over the values 10^−7^, 10^−6^, 10^−5^, and 10^−4^. In these non-mixed runs, direct interactions are only between neighboring grid boxes, defined as the eight grid boxes surrounding a focal box. The model was also run in a well-mixed mode, in which the interacting box is drawn randomly from the entire grid, so that each box is equally close to every other box, removing spatial effects. For the well-mixed scenarios, the EDiff was always set to 0.5. These scenarios were run both with and without mutation enabled. We also ran the model with only two types, *CNP* (generalist producers) and *cnp* (cheaters). These runs did not include mutation.

We ran the model for 35,000 h for the full genotype set and 8,000 h for the two-typeset and for the full set in the case with EConstit = 10^−4^. For the 35,000 h runs, we ran five replicates both with and without mutation. For the scenario with EConstit = 10^−5^ and E Diff = 10^−2^, we ran six additional replicates.

### Model outputs

The outputs of the model are population density by genotype, diversity, and depolymerization of C, N, and P. Densities were computed as the number of microbes over the area of the grid, which is 10,000 μm^2^. Diversity was calculated as $1-\sum p_{i}^{2}$, where *p_i_* is the proportion of genotype *i* in the community. This quantity is bounded by 0 and 1, and higher values represent greater diversity. Nutrient depolymerization is the amount of substrate that is converted into product per hour, whether or not this product is taken up and used by microbes. Diversity and nutrient depolymerization were averaged over the last 10,000 model hours of all replicate runs.

### Spatial analysis

To test for spatial associations between microbial types, we used spatial statistics on independent samples of the grid outputs. Grid outputs that are close in time cannot be considered independent because of temporal autocorrelations. Therefore, we calculated the time required for these autocorrelations to disappear and used these intervals as a basis for sampling the grid. The number of time steps required for two time slices to be considered independent is *N_t_* = (1+*r_t_*)/(1−*r_t_*), where *r_t_* is the correlation between time slices, computed as the proportion of grid boxes that retained the same value at times *t* and *t* + 1. This value tended to increase over the course of a run, so one cannot assume that it is constant. We took the first time slice at *t* = 1, and then additional slices moving in steps of *N_t_*, where *t* is in units of 1,000 h, and *N_t_* is computed dynamically at each time slice and rounded up to the next integer.

At each independent time point, we analyzed the spatial associations of the microbial types using a multi-way “join-count” analysis. This analysis “counts” the number of “joined” (neighboring) cells of a pair of focal types and compares this number to the expected count assuming a purely random spatial distribution, yielding a *z*-statistic for each pair of types (Cliff and Ord, 1973, 1981). We used the “joincount.multi” and “cell2nb” functions in the “spdep” package in R (Bivand, 2012) to perform the calculations.

The *z*-statistics from the join-count analysis were squared and then summed over all time slices for all replicates of a given scenario to give a χ^2^ statistic. We also computed a total χ^2^ statistic summed over all scenarios. These statistics were then compared to a χ^2^-distribution with degrees of freedom equal to the number of independent time slices analyzed for that scenario (or over all scenarios) in which the pair was present. As there are 45 pairs (including empty boxes), we applied the Bonferroni correction to get a cutoff of 0.05/45 = 0.0011. For each pair, we computed the average *z*-statistic over all cases for which there was a significant positive association, in order to get a score for the strength of the association.

The default implementation of the “cell2nb” function allows one to analyze a grid only in the case where each cell is considered “joined” to only its nearest neighbors. However, this spatial scale was too small to yield significant positive correlations between different types. We modified this function to join all cells in a neighborhood with a radius of any length. We ran the analysis with neighborhood radius lengths of 1, 2, 3, 4, and 5 grid boxes. As the relative values of the *z*-scores did change depending on the radius length, we averaged the *z*-scores for each pair over all radius lengths. Since smaller neighborhoods are contained within larger neighborhoods, this method implicitly weights closer neighbors more than farther neighbors by 1/*x*, where *x* is the neighborhood radius. We used these average *z*-scores to rank the pairs by strength of association.

To quantify the degree of complementarity between pairs of types, we calculated a complementation score as the number of enzyme loci with different alleles. For example, the pair (*CNp*, *Cnp*) has a score of 1, because it has the same alleles at the C and P loci and different alleles at the N locus. Across all pairs of types, we tested the correlation between this complementation score and the strength of the spatial association based on *z*-scores from the join-count analyses.

## Results

In both the two-type and eight-type models, under low diffusion and constitutive production, producers dominated the community. As constitutive enzyme production and enzyme diffusion increased, cheaters made up a larger proportion of the community. When EConstit was increased to 10^−4^, the high cost of production caused the community to go extinct. Community extinction also occurred in the well-mixed scenarios. In the scenario with high constitutive production (EConstit = 10^−5^) and diffusion (EDiff = 10^−2^), the two-type model resulted in extinction, but in the eight-type model, the community survived in 64% of replicates.

### Diversity and community composition

**Figure 1:**
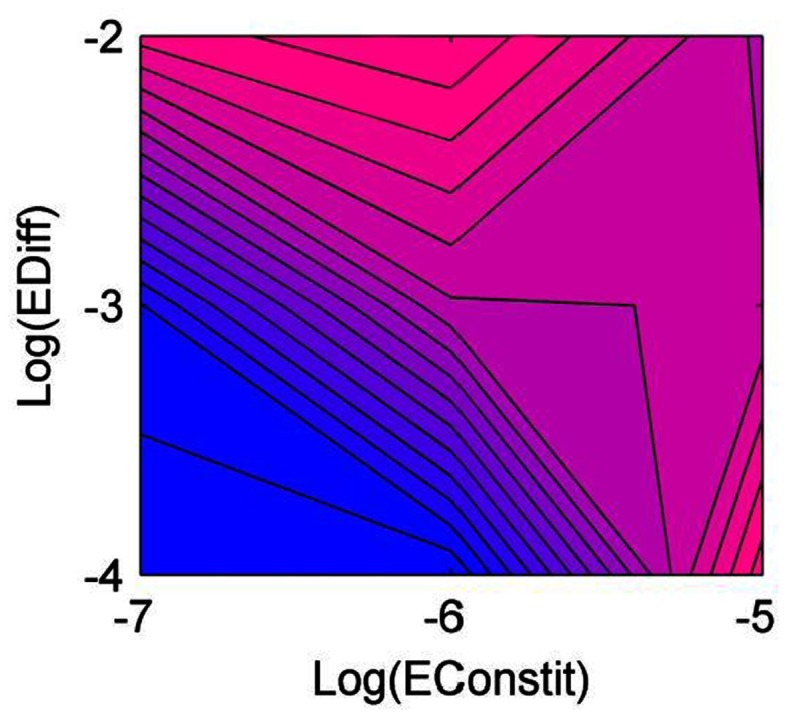
**Diversity of the community as a function of diffusion rate (EDiff) and constitutive enzyme production rate (EConstit), averaged over all replicate runs**. Blue represents low diversity, and pink high diversity.

**Figure 2:**
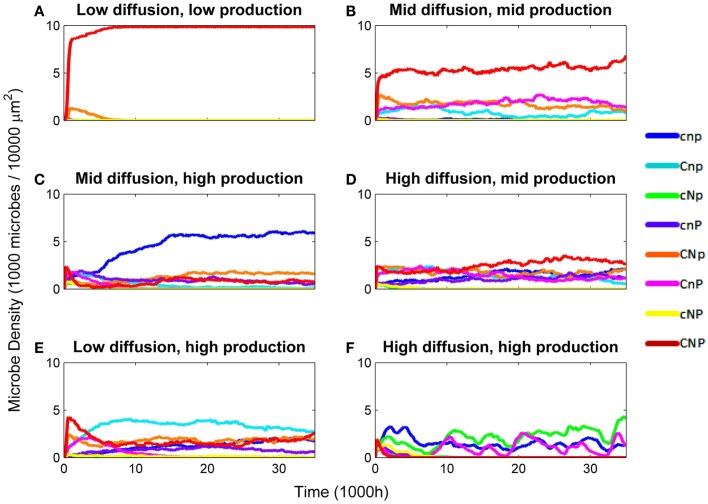
**Density of microbes by genotype over various combinations of diffusion rate (EDiff) and constitutive enzyme production rate (EConstit)**. Densities are in 1,000 microbes/10,000 μm^2^. **(A)** EDiff = 10^−4^, EConstit = 10^−7^. **(B)** EDiff = 10^−3^, EConstit = 10^−6^. **(C)** EDiff = 10^−3^, EConstit = 10^−5^. **(D)** EDiff = 10^−2^, EConstit = 10^−6^. **(E)** EDiff = 10^−4^, EConstit = 10^−5^. **(F)** EDiff = 10^−2^, EConstit = 10^−5^.

Diversity was lowest when constitutive production and diffusion were both low (Figure 1). Generalist producers (*CNP*) dominated, driving all other types to extinction (Figure 2A). Under intermediate constitutive production and diffusion, although generalist producers (*CNP*) were still most common, they only made up approximately half of the community, with the remainder including the three other C-producers, *CNp*, *CnP*, and *Cnp* (Figure 2B). Increasing constitutive production while holding diffusion constant caused a shift in the community composition, but had little effect on diversity (Figure 1). Cheaters (*cnp*) made up half of the community, and types *CNP*, *CNp*, and *cnP* were also moderately successful (Figure 2C). The highest diversity was found under intermediate constitutive production and high diffusion (Figure 1). All types other than *cNP* and *cNp* were able to persist, and at relatively even frequencies, but with *CNP* at the highest frequency (Figure 2D). Diversity also peaked under high constitutive production and low diffusion, showing that high diversity could be maintained either by high diffusion or high constitutive production, but not both (Figure 1). The mixture of types at this scenario included more of types *cnp* and *Cnp*, because high constitutive production favors cheating more than high diffusion (Figure 2E).

Diversity was intermediate under high constitutive production and diffusion, which favor cheaters and coalitions (Figure 1). Competition from cheaters drove the community to very low densities, with most types going extinct. However, as densities declined, competition from cheaters was relaxed, and the surviving types were able to rebound in some cases. The specific types that survived this bottleneck were determined randomly, but in order to survive, the community must include a set of types that produce all three nutrients, for example, types *CnP* and *cNp* (Figure 2F). Cheaters (*cnp*) were nearly always successful in this scenario, due to the high diffusion and constitutive production. Density-dependent competition from cheaters caused the total density to cycle. Mutation has the ability to reintroduce types lost during the bottleneck, so diversity tended to be higher under mutation in this scenario.

**Figure 3:**
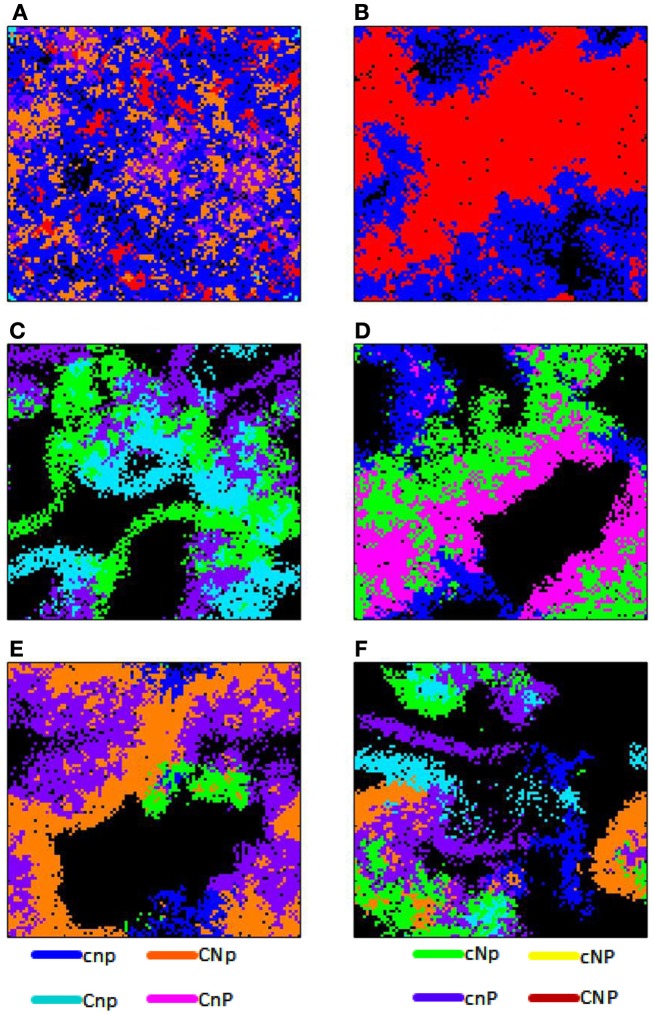
**Spatial grid at a time slice for various scenarios**. In all figures, constitutive enzyme production is high (EConstit = 10^−5^). **(A)** Enzyme diffusion is intermediate (EDiff = 10^−3^), *t* = 34,000 h. **(B–D)** Show different replicates with high enzyme diffusion (EDiff = 10^−2^). **(B)** Types CNP (red) and (cnp) blue, *t* = 20,000 h. **(C)** Types *CnP* (pink), *cNp* (green), and *cnp* (blue), *t* = 21,000 h. **(D)** Types Cnp (light blue), cNp (green), and cnP (purple), *t* = 25,000 h. **(E)** Types CNp (orange), cNp (green), cnP (purple), and cnp (blue), *t* = 27,000 h. **(F)** Types CNp (orange), Cnp (light blue), cNp (green), cnP (purple), and cnp (blue), *t* = 27,000 h.

**Figure 4:**
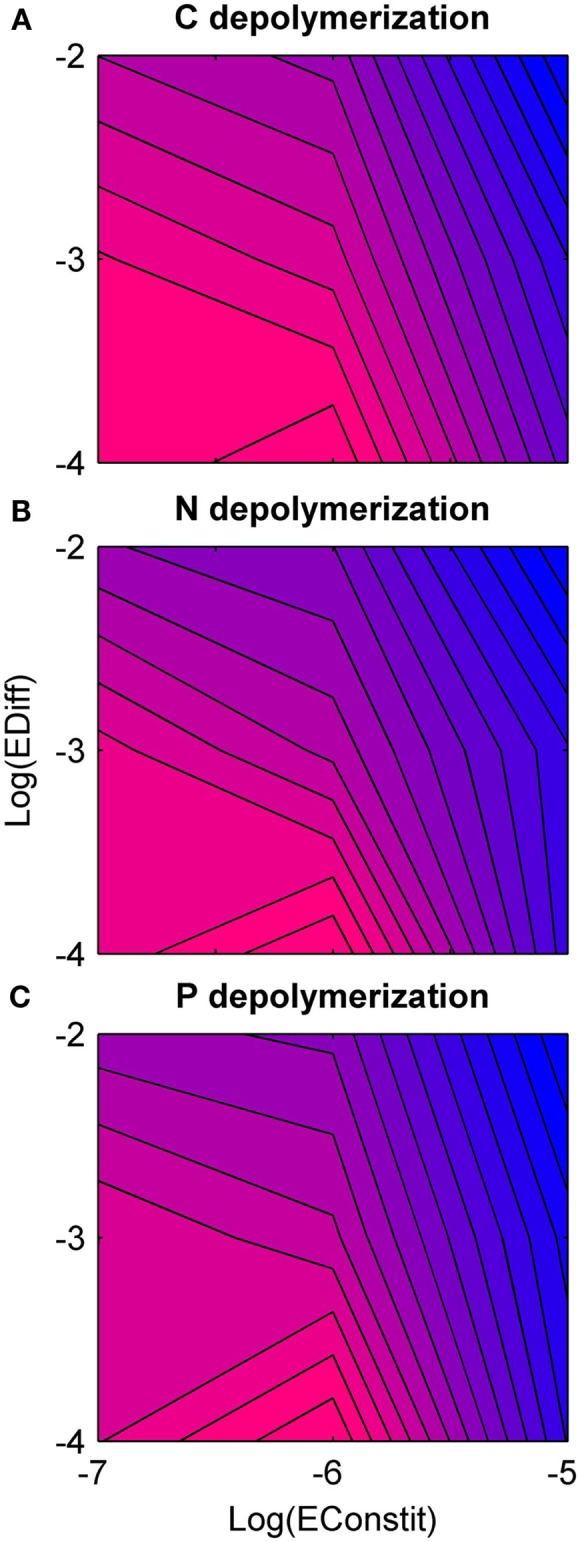
**Rate of nutrient depolymerization as a function of diffusion rate (EDiff) and constitutive enzyme production rate (EConstit) for each of the three nutrients, averaged over all replicate runs**. Blue represents low rates of depolymerization, and pink high rates. **(A)** Carbon depolymerization. **(B)** Nitrogen depolymerization. **(C)** Phosphorous depolymerization. Across all three nutrients, the depolymerization rate is highest for intermediate production and low diffusion, and lowest for high production and high diffusion. The depolymerization rate decreases with the diffusion rate over the domain studied.

The variability of the outcomes also increased with diffusion and constitutive production, and the last scenario was much more variable than the others, due to the bottleneck. Figures 1 and 4 show results averaged over all replicate runs, and Figures 2 and 3 show representative individual runs.

### Spatial associations and coalitions

The strongest spatial associations were between microbes of the same type (Table 1). All of these associations were statistically significant at all spatial scales. C-enzyme producers were more strongly autocorrelated, as indicated by a significant correlation between C production and rank (*r* = −0.87, *p* = 0.048). However, there was no association between N- or P-enzyme production and the degree of autocorrelation.

**Table 1:** **Ranking of spatial autocorrelation between pairs of the same genotype with average *z*-score**.

Types	Score
CNp	8.86*
CNP	8.29*
Cnp	6.98*
CnP	6.86*
cnP	6.64*
cnp	6.48*
cNp	6.44*
cNP	6.21*

*Denotes statistical significance with Bonferroni correction at all scales.

Associations involving five pairs of different types were significant when summed across all scenarios at the 5-μm radius scale (Table 2). Fewer were significant at shorter distances, with none significant at the 1-μm radius scale. The correlation between average *z*-score and complementation score was significant (*r* = 0.71, *p* = 0.035), indicating that complementary types were more strongly associated.

**Table 2:** **Ranking of spatial association between pairs of different genotypes, with average *z*-score and complementation score, defined as the number of loci at which they have different alleles**.

Types	Score	Comp.
cnP,CNp	0.62*	3
CnP,cNp	0.40*	3
cNp,Cnp	0.34*	2
CNP,cnp	0.26*	3
cNP,Cnp	0.23*	3
CnP,CNp	0.20	2
cNP,CNp	0.20	2
CNp,cnp	0.17	2
cNP,CnP	0.14	2
cnP,Cnp	0.10	2
CNP,cNp	0.09	2
CNp,cNp	0.08	1
cNP,cNp	0.08	1
CNp,Cnp	0.07	1
CNP,cNP	0.07	1
Cnp,cnp	0.06	1
CnP,cnP	0.06	1
cNP,cnP	0.05	1
CnP,cnp	0.05	2
CNP,CnP	0.05	1
cNp,cnp	0.05	1
CNP,cnP	0.05	2
cnP,cNp	0.04	2
cNP,cnp	0.03	2
CnP,Cnp	0.02	1
cnP,cnp	0.02	1
CNP,Cnp	0.02	2
CNP,CNp	0.02	1

*Denotes statistical significance with Bonferroni correction at the 5 μm scale.

Although associations between complementary types could be statistically detected when averaged across all scenarios, coalitions only played a major role in scenarios with high constitutive production. Under intermediate diffusion, the coalition between *CNp* and *cnP* was moderately successful, despite the high density of cheaters (Figure 2C). Colonies of these two types were strongly associated, as were types *CNP* and *cnp* (Figure 3A). The scenario with high diffusion and constitutive production (EDiff = 10^−2^, EConstit = 10^−5^) displayed uniquely variable outcomes due to a bottleneck caused by density-dependent competition from cheaters. These variable outcomes highlight how mutualistic, competitive, and parasitic interactions shaped the spatial patterns of the community.

When only types *CNP* and *cnp* survived, the producers formed dense aggregations due to their facilitative interactions, whereas cheaters were more diffuse due to their competitive relationship with each other, and clung to the edges of producer colonies due to their parasitic relationship with them (Figure 3B; Movie S1 in Supplementary Material). Densities of both types cycled due to negative density-dependent fitness of the producer and delayed tracking of producer density by the cheater.

When types *Cnp*, *cNp*, and *cnP* survived, they formed a three-way coalition (Figure 3C; Movie S2 in Supplementary Material). In this case, the relationship is opposite to that described above, in that microbes of the same type inhibit each other’s growth due to competition for the nutrients for which they cannot produce enzymes, but facilitate the growth of the other types by providing their complementary enzymes. This led to coalitional colonies of interwoven filamentous shapes.

When types *CnP* and *cNp* survived, they formed a successful coalition (Figures 2F and 3D; Movie S3 in Supplementary Material). The relationship between these types includes both mutualistic and parasitic aspects. Both produce the complementary enzymes required by the other type, causing them to facilitate each other’s growth. However, there is an inherent asymmetry, because one produces only one enzyme and the other two. The net impact of *cNp* on the fitness of *CnP* was positive at low density, but negative at high density, when competition for C became overwhelming, leading to cycling. Types *CNp* and *cnP* have a similar relationship and formed similar spatial patterns (Figure 3E; Movie S4 in Supplementary material).

The outcome with the highest diversity included five types, *CNp*, *Cnp*, *cNp*, *cnP*, and *cnp*, and displayed patterns of both mutualistic and parasitic interactions (Figure 3F; Movie S5 in Supplementary Material). Type *CNp* had a higher growth rate and was more independent relative to other types, and so formed larger colonies, whereas the single-enzyme producers formed thinner, more filamentous colonies because of competitive interactions with their own type. Density-dependent effects also led to cycling of *CNp* in this scenario.

### Nutrient depolymerization

Overall, holding constitutive production constant, nutrient depolymerization was highest for low diffusion (Figure 4). Under low diffusion, depolymerization was highest under intermediate constitutive production, and under intermediate to high diffusion, depolymerization was highest under low constitutive production (Figure 4). A negative correlation was found between diversity and nutrient depolymerization of −0.45, −0.29, and −0.38 for C, N, and P, respectively. The *p*-values for these correlations were 0.020, 0.051, 0.030. This indicates weak significance for C and P, although if a Bonferroni correction with *n* = 3 is applied, none are significant.

In comparison with the two-type model, the eight-type model showed reduced depolymerization rates, especially for N and P. This effect was strongest under high constitutive production and low diffusion. In the two-type model, producers were able to dominate cheaters in this scenario. However, in the eight-type model, a diverse mix of types persisted, including a relatively high frequency of *Cnp* (Figure 2E), accounting for the low depolymerization of N and P. This effect was reversed under high constitutive production and diffusion, because in this case, the two-type model went extinct, whereas the eight-type model was able to survive the bottleneck caused by the initial crash in some replicates. Therefore, its average nutrient depolymerization rates were much higher than the two-type model.

## Discussion

We have modeled the enzyme-mediated depolymerization of nutrients by microbes as a public goods game in a diverse community of microbes. Our model differs from previous models in several important ways. While many models specify fitness payoffs of an evolutionary game exogenously (Durrett and Levin, 1994; Kerr et al., 2002; Hauert and Doebeli, 2004; Gore et al., 2009; Wakano et al., 2009), this model mechanistically models the production and action of enzymes by the microbes, and allows the game dynamics to emerge from them. Furthermore, rather than arbitrarily specifying the efficiency of resource capture (as do Gore et al., 2009) or the size of the interaction neighborhood (as do Wakano et al., 2009), we simply allow enzymes to diffuse through the environment. By increasing the diffusion rate, we reduce the efficiency of resource capture for enzyme producers, thus reducing the benefits of enzyme production for producers and increasing the amount of product available for cheaters. For this reason, faster diffusion benefits cheaters at the expense of producers (Allison, 2005).

This model builds on the previous one by Allison (2005) by the addition of multiple types of microbes, which may or may not produce enzymes for the three types of nutrients in the model. Thus there are two (not completely orthogonal) axes on which types vary, the number of enzymes they produce (from 0 to 3) and the types of enzymes they produce (C, N, or P). Most models of public goods games include only two types of agents, producers and cheaters. Some models of allelopathy include three types of agents, which can lead to rock-paper-scissors dynamics that allow for the maintenance of diversity (Durrett and Levin, 1997; Kerr et al., 2002). However, real microbial communities are highly diverse, as are the enzymes produced by them and the substrates they hydrolyze. While this model does not seek to model the full diversity of a real system, increasing the number of types from two to eight allows us to gain some heuristic insight into the relationship between the diversity of enzyme production strategies and three important biological properties: diversity, social interactions, and nutrient depolymerization.

### Diversity

Diversity was highest under intermediate conditions, specifically relatively fast diffusion (but not well-mixed), and intermediate constitutive production. Under these conditions, most types were able to coexist at relatively even frequencies. The intermediate level of constitutive production maintained a balance between producers and cheaters, and the fast diffusion allowed for the sharing of enzymes between complementary types. Diffusion rate is a function of both the size of the enzymes and the physical environment. The diffusion rates considered in this study are much slower than published diffusion coefficients in liquid media (Vetter et al., 1998; He and Niemeyer, 2003). They are intended to model diffusion in complex soils or sediment matrices, in which diffusion is slowed due to complex pore structures (Moldrup et al., 2001). Therefore the model predicts that environments allowing for an intermediate rate of diffusion should have higher diversity than either well-mixed environments or ones where diffusion is highly constrained.

Consistent with our model predictions, empirical studies show that differences in diffusion rates can influence microbial diversity and community composition. In a soil microcosm study, Carson et al. (2010) found that bacterial diversity increased at lower water potential, indicative of reduced soil pore connectivity and diffusion rates. Bacterial diversity and evenness also increased systematically as water content declined across a gradient of 29 soils sampled in the field (Zhou et al., 2002). Competition experiments under controlled conditions further suggest that rates of diffusion influence coexistence among bacteria. In soil microcosms, coexistence between *Ralstonia* and *Sphingomonas* bacteria increased with decreasing soil water potential (Treves et al., 2003). Another laboratory experiment found that two competing *Pseudomonas putida* strains coexisted on diffusion-limited agar plates but not in liquid culture (Dechesne et al., 2008). Thus there is good empirical support for the prediction that increasingly well-mixed conditions reduce the diversity of bacterial communities. However, our model also predicts that very low rates of diffusion could reduce diversity by selecting against cheaters that depend on diffusion to access enzyme reaction products. Since most prior studies have focused on relatively high diffusion environments, additional experiments should be conducted to test this prediction.

### Social interactions

In the domain of social interactions, our model reveals an alternative strategy to generalist production and cheating: the formation of coalitions between complementary types. Our model assumes that if a microbe has the potential to make an enzyme, it must do so at constitutive levels or more, making the generalist producer strategy (*CNP*) inherently costly. Under low constitutive production, these costs are small, generalist producers dominate, and neither coalitions nor cheaters are observed. However, as constitutive production increases, enzyme costs reduce the competitive ability of generalist producers. While this level of public goods production favors the evolution of cheaters, it also favors coalitions that reduce the costs of enzyme production by allowing microbe types to obtain resources through the activity of complementary types. The same conditions that favor cheaters also favor coalitions because mechanistically, coalitions can be thought of as mutual “cheating,” since both types take advantage of the enzymes produced by the other. Interactions between complementary types can be mutualistic, if they facilitate each other’s growth, or parasitic, if one benefits at the expense of the other. We found that complementary types were spatially associated regardless of whether their interaction was mutualistic or parasitic, but the spatial association was stronger for mutualistic interactions. Therefore, if different microbe types are spatially associated, they should produce complementary enzymes, and their interaction is probably mutualistic, but need not be.

Coalitions were most important under high constitutive production and diffusion. Under these conditions, the two-type community was not stable, and crashed due to cheating. However, the eight-type community was able to survive the bottleneck of the initial crash and rebound in some replicates, albeit with reduced diversity. In some cases, the community even entered a cyclic state of repeated crashes and rebounds. This behavior is due to the density-dependence of microbe fitness, since producers have the advantage at low density, but cheaters the advantage at high density. More abstract models of evolutionary games have also shown that the addition of an advantage to cooperators at low densities allows for coexistence in a public goods game (Durrett and Levin, 1994; Wakano et al., 2009). These theoretical predictions are also supported by experimental evidence from the yeast *Saccharomyces cerevisiae*, which produces an extracellular enzyme that hydrolyzes the disaccharide sucrose into glucose. By varying the density of cells, Greig and Travisano (2004) showed that cooperators have higher fitness at low densities, but that cheaters have higher fitness at high densities. Since cheaters rely on the enzymes produced by cooperators, they are not able to survive at low densities, but enzyme producers are self-sufficient, and so are able to survive even at low density. However, at high densities, the enzyme is plentiful, and the cheaters are able to outcompete the cooperators because they do not pay the cost of enzyme production.

In addition, the high diffusion and constitutive productions scenario revealed differences in the emergent spatial patterns of different microbial associations. Fully cooperative interactions led to a dense, highly aggregated pattern with high autocorrelation, as observed for *CNP–CNP* interactions. In contrast, fully competitive interactions led to a dispersed pattern with lower autocorrelation, as observed for *cnp–cnp*. Interactions between complementary types include aspects of both cooperation and competition, and therefore produced more complex spatial patterns.

### Nutrient depolymerization

Social interactions could complicate the relationship between microbial diversity and ecosystem function (Nielsen et al., 2011). Nutrient depolymerization was highest for low diffusion and low to intermediate constitutive production. In the absence of competition from cheating, depolymerization increased with increasing diffusion and constitutive production (Allison, 2005), due to the fact that high constitutive production directly increases the quantity of enzymes produced, and high diffusion reduces enzyme saturation by spreading enzymes from areas of high concentration to areas of low concentration. However, these conditions also favored cheaters and coalitions of intermediate types in competition with generalist producers, and when these effects were accounted for, the net effects of high constitutive production and diffusion were reversed, reducing nutrient depolymerization.

By comparing nutrient depolymerization rates between the two-type and eight-type models, we found that nutrient depolymerization for all nutrients tended to be lower in the more diverse model (except under high constitutive production and diffusion where the two-type community went extinct). This effect may seem counterintuitive, because it is often assumed that increasing diversity will increase the rate of resource use due to niche complementarity between types (Tilman, 1999; Tilman et al., 2001). However, when social interactions are also considered, the effect of diversity may also be reversed, reducing nutrient depolymerization.

The reduction in depolymerization was strongest for P, intermediate for N, and weakest for C. This pattern was due to C-limitation of microbial growth in the model imposed by a higher stoichiometric demand for C relative to the substrate supply. Therefore C-only producers could acquire the most valuable resource while paying less cost of enzyme production than generalist producers. Competition from C-only producers reduced the density of generalist producers, and consequently N- and P-depolymerization were reduced relative to C-depolymerization. Therefore a prediction of our model is that N- and P-depolymerization are reduced more by competition than C-depolymerization, a prediction that could not be made by a simpler two-type model. Furthermore, this prediction is general and not restricted to C-limitation. Cheating is constrained for the most limiting nutrient, so the less limiting a nutrient is biologically, the more its depolymerization will be reduced by social interactions.

In this study, we showed that increasing diversity from two to eight types reduced nutrient depolymerization rates due to social interactions. However, real microbial communities contain thousands of taxa, although at this time it is unknown how much functional diversity is represented by this taxonomic diversity. Therefore, an important challenge for future work is to understand how social interactions influence nutrient dynamics at the high levels of microbial diversity observed in real ecosystems. Understanding these scaling rules could improve our ability to predict carbon and nutrient cycling processes driven by complex microbial communities.

## Conflict of Interest Statement

The authors declare that the research was conducted in the absence of any commercial or financial relationships that could be construed as a potential conflict of interest.

## Supplementary Material

The Supplementary Material for this article can be found online at http://www.frontiersin.org/Terrestrial_Microbiology/10.3389/fmicb.2012.00338/abstract

Supplementary Movie S1 **Cycling of generalist producers and cheaters**. Only three types survive the initial bottleneck: generalist producers (red), cheaters (blue), and P-only producers (purple). When densities are low, generalist producers are capable of very high growth rates because they catalyze their own growth, and so form large aggregations. However, their high cost makes them poor competitors at high densities, and they are outcompeted by cheaters. Once density drops due to the cheater load, producers rebound, leading to cyclical behavior (http://dx.doi.org/10.6084/m9.figshare.92321).

Supplementary Movie S2 **Three-way coalition of single-enzyme producers**. Only three types survive the initial bottleneck: C-only (light blue), N-only (green), and P-only (purple). These types compete with their own type for the resources of enzymes they cannot produce, but catalyze each other’s growth by providing their complementary enzymes. This leads to a spatial pattern in which the shapes of colonies maximize their perimeter, and different types are highly interwoven. Mutations can be seen when small colonies of other colors appear, but none of these are able to invade, indicating that this community is stable to invasion. This is the same replicate as Figure 3D (http://dx.doi.org/10.6084/m9.figshare.92322).

Supplementary Movie S3 **Asymmetric coalition between a two-enzyme producer and a one-enzyme producer**. Only three types survive the initial bottleneck: *CnP* (pink), *cNp* (green), and *cnp* (blue). *CnP* and *cNp* catalyze each other’s growth at low density, but *cNp* becomes parasitic on *CnP* at high densities, leading to complex spatial patterns and cycling (http://dx.doi.org/10.6084/m9.figshare.92323).

Supplementary Movie S4 **Four types survive the initial bottleneck: CNp (orange), cNp (green), cnP (purple), and cnp (blue)**. *CNp* and *cnP* complement each other, forming a mutualistic coalition at low densities, but at high densities, competition from the other types causes collapse of the *CNp* population, nearly leading to extinction. Once densities are low enough, *CNp* rebounds quickly, leading to cycling (http://dx.doi.org/10.6084/m9.figshare.92325).

Supplementary Movie S5 **High diversity. In this scenario, five type survive the bottleneck: CNp (orange), Cnp (light blue), cNp (green), cnP (purple), and cnp (blue)**. This scenario includes the mutualistic interactions between single-enzyme producers as in Movie S2, the asymmetric coalition between *CNp* and *cnP* in Movie S4, and competition from cheaters, leading to a complex set of interactions. *CNp* is able to grow much faster than other types because it is more independent and catalyzes its own growth. This community is stable to invasion by other types due to mutation and represents the highest diversity outcome of this scenario (http://dx.doi.org/10.6084/m9.figshare.92324).
